# Bilateral Gum Chewing as Proprioceptive Sensorimotor Re-Education for Patients with Masticatory Muscle Weakness Presenting with Orofacial Pain and Perceived Occlusal Change: A Technical Note with a Retrospective Case Series

**DOI:** 10.3390/bioengineering13070826

**Published:** 2026-07-17

**Authors:** Hyun-Jeong Park, Jong-Mo Ahn, Young-Jun Yang, Ji-Won Ryu

**Affiliations:** Department of Oral Medicine, School of Dentistry, Chosun University, Gwangju 61452, Republic of Korea; rosephj81@chosun.ac.kr (H.-J.P.); jmahn@chosun.ac.kr (J.-M.A.); jyyy15413@chosun.ac.kr (Y.-J.Y.)

**Keywords:** G-SCORE, bilateral gum chewing, masticatory muscle, orofacial pain, occlusion, proprioception, periodontal mechanoreceptors, trigeminal mesencephalic nucleus, sensorimotor re-education, temporomandibular disorders

## Abstract

Within the Diagnostic Criteria for Temporomandibular Disorders, masticatory myalgia is framed around muscle hyperactivity, yet a recognizable subgroup shows the opposite profile—low muscle tone, fatigue, masticatory pain, and a persistent sense that the bite no longer fits, without structural dental change—and responds poorly to conventional conservative care. We introduce G-SCORE (Gum-chewing-mediated Sensorimotor Calibration of Occlusion through Rhythmic Exercise), a proprioceptive recalibration strategy delivered as a bilateral gum-chewing (BGC) protocol: two equal halves of one sugar-free gum chewed simultaneously and symmetrically on both posterior segments in short, patient-titrated sessions, delivering balanced, rhythmic, low-load input to the trigeminal sensorimotor system. In a retrospective series of 41 patients, we characterized within-subject changes in pain, mandibular mobility, and occlusal parameters. Masticatory pain fell from 5.0 ± 2.9 to 0.7 ± 0.9 (0–10; *n* = 27; *p* < 0.001; r = 0.87), with 85% achieving a ≥2-point reduction. Occluding tooth positions and mouth opening also recovered; in an anterior open-bite subgroup, overbite improved (*n* = 6; *p* = 0.031). The observed changes were dominated by pain relief and restored bilateral occlusal contact rather than by raw force—a pattern more compatible with sensorimotor recalibration than with muscle hypertrophy. This low-cost, non-invasive technique may help selected patients avoid irreversible occlusal treatment.

## 1. Introduction

The masticatory system depends on the coordinated action of the masseter, temporalis, and medial and lateral pterygoid muscles to generate occlusal force, sustain mandibular posture, and execute the chewing cycle, with the masseter acting as the principal force-generating elevator [1,2]. Within the Diagnostic Criteria for Temporomandibular Disorders (DC/TMD) framework, masticatory myalgia is defined principally by pain on palpation or function [3].

In day-to-day specialist practice, however, a recognizable subgroup presents with the opposite functional profile: low palpable muscle tone, fatigue at sub-maximal chewing loads, and a pervasive complaint that the bite “no longer fits,” in the absence of any structural dental or restorative change that would account for it. A characteristic feature of these patients is that they respond poorly to the conventional conservative regimen used for muscular temporomandibular disorders, in which generally only reversible, conservative strategies are recommended [4,5]. Although TMD-related pain tends to improve to some extent regardless of the modality used, a substantial proportion of patients progress to chronicity or report persistent symptoms [5], and exercise- and resistance-based programs remain limited by undefined protocols and low-quality evidence [6]. In the subgroup described here, this dissociation is pronounced: simple home care and self-management lower the resting or background pain in the early phase—consistent with the analgesic effect of conservative muscle therapy [7]—yet the patients paradoxically report worsening function rather than recovery, namely increasing fatigue on chewing, a growing sense of occlusal discomfort or instability, and an increase in pain specifically on loading and function. In other words, the pain that responds to rest is decoupled from the functional weakness, which persists or deteriorates, a refractory, persistent pattern conceptually aligned with the recently proposed notion of treatment-refractory masticatory dysfunction [8]. Masticatory muscle weakness—rather than hyperactivity—thus emerges as the dominant feature; it is frequently uncovered during the diagnostic work-up rather than volunteered as the presenting concern, and the persistence of functional and occlusal complaints after standard conservative care is itself a diagnostic clue to this phenotype.

Jaw sensorimotor function and bite force decline with age, and the masseter, as a skeletal muscle, is subject to atrophy and sarcopenic change [8,9], a decline that is closely linked to oral hypofunction, frailty, and sarcopenia in older adults [10,11]. Yet weakness of the masticatory muscles is not confined to the elderly. It also follows chronic pain-related disuse and neuromuscular disease—for example, reduced occlusal force in Duchenne muscular dystrophy [12] and patient-reported mastication difficulty in McArdle disease [13]—as well as intramuscular botulinum-toxin treatment and masseter-reduction procedures, which measurably reduce bite force and masseter thickness [14], and prolonged splint wear. In each of these settings the muscles are under-loaded, and the patient often adopts a guarded, unilateral, low-amplitude chewing pattern that further reduces and unbalances the functional afferent input arising from the dentition and the jaw muscles [15].

Chewing-based exercise has an established, if heterogeneous, evidence base. Gum-chewing and bilateral chewing programs increase maximum occlusal force and, in some studies, masseter thickness in community-dwelling older adults [1,2,16,17], and structured oral-function training improves tongue pressure and bite force in denture wearers [18]. Notably, gum-chewing training can increase occlusal force and occlusal contact area without a measurable change in masseter thickness, indicating that a substantial part of the functional benefit is neuromuscular rather than hypertrophic [16], and even with isometric loading a rise in voluntary bite force may reflect motor learning rather than true strengthening [19]. Most protocols, however, are designed either for healthy participants or for frail older adults with oral hypofunction [10,11], are frequently unilateral, and target force gain as the primary endpoint; none are tailored to the orofacial-pain patient in whom masticatory weakness, myalgic pain, and a perceived occlusal disturbance coexist.

This report has two aims. First, we introduce G-SCORE (Gum-chewing-mediated Sensorimotor Calibration of Occlusion through Rhythmic Exercise) as a proprioceptive sensorimotor recalibration strategy developed for this specific subgroup, articulate its neurophysiological rationale as proprioceptive sensorimotor re-education rather than strengthening, and describe the operational bilateral gum-chewing (BGC) protocol through which the strategy is delivered. Second, we characterize within-subject changes in pain, mandibular mobility, and occlusal parameters in a retrospective series of 41 consecutive patients managed under this strategy, and we describe the pattern of response, dominated by pain relief and recovery of bilateral occlusal contact rather than by raw force. The technique requires no equipment beyond commercially available sugar-free gum and is reproducible by general dental practitioners and orofacial-pain specialists.

## 2. Materials and Methods

### 2.1. Study Design and Patients

#### 2.1.1. Study Design

This was a single-center, retrospective chart review of consecutive patients managed with the G-SCORE strategy (delivered as the BGC protocol) in the Department of Oral Medicine, Chosun University Dental Hospital, between January 2020 and June 2025.

#### 2.1.2. Eligibility and Diagnostic Criteria

Eligible patients met all of the following criteria. (i) Diagnostic criterion—a diagnosis of temporomandibular disorder according to the Research Diagnostic Criteria for Temporomandibular Disorders (RDC/TMD) or the Diagnostic Criteria for Temporomandibular Disorders (DC/TMD). (ii) Phenotypic criterion—concurrent masticatory muscle weakness identified during the diagnostic work-up on standardized clinical examination by an orofacial-pain specialist, operationally defined as the presence of at least two of the following features: reduced palpable masseter or temporalis tone; weak or delayed masseter and temporalis contraction on voluntary clenching; fatigue at sub-maximal chewing loads; reduced masticatory muscle endurance on repeated function; or a guarded, low-amplitude, unilateral chewing pattern. The clinical phenotype was established by uniform expert examination in all included patients; specialist palpation and clenching-based evaluation reliably identify reduced masticatory muscle activation in this setting and are the diagnostic reference used for patient selection. B-mode masseter ultrasonography (LOGIQ P9 R3, GE Healthcare, Milwaukee, WI, USA) [1,2] and quantitative bite-force measurement were performed when clinically indicated and when equipment was available (Section 3.1) and were used to corroborate the clinical phenotype rather than to establish it, so that phenotype definition remained uniform across the cohort while the availability of objective corroboration varied by patient. Although the referral aetiologies were heterogeneous (Section 3.1), all included patients converged on the same operational category of reduced masticatory muscle activation with pain on function and a perceived occlusal disturbance in a structurally stable dentition. (iii) Adherence criterion—documented performance of the prescribed BGC protocol for a minimum of four weeks.

Exclusion criteria were an orofacial infectious disease, a neurological disorder in the systemic history, intervention performed for less than four weeks, records insufficient for analysis (missing chart, imaging, or bite-force data where relevant), and any structural dental, restorative, or prosthetic change sufficient to explain a perceived bite change (which would place the patient outside the phenotype of interest).

#### 2.1.3. Patient Characteristics

Forty-one consecutive patients met these criteria (34 women, 7 men; mean age 39.5 ± 18.3 years, range 15–74; 9 patients [22%] aged ≥60). Referral contexts comprised temporomandibular joint osteoarthritis with occlusal instability or myalgia (*n* = 26), post-splint or appliance management (*n* = 7), post-masseter botulinum-toxin pain (*n* = 5), and single cases following facial trauma, temporomandibular joint arthroplasty, and masseter-reduction surgery. Where cone-beam computed tomography (CBCT; Carestream Health, Inc., Rochester, NY, USA) was performed (33 patients), degenerative joint change was documented in 32 cases. Masseter ultrasonography, performed in 12 patients, showed masticatory muscle weakness in 7. A baseline complaint of occlusal change was recorded in 26 patients. Because the analysis was retrospective, the availability of each outcome varied across patients; the paired sample size is therefore reported separately for every variable.

#### 2.1.4. Ethical Considerations

The study was approved by the Institutional Review Board of Chosun University Dental Hospital (Approval Number: CUDHIRB 2509 002). As this was a retrospective chart review, all patient data were de-identified and anonymized prior to analysis, and the requirement for individual informed consent was waived in accordance with IRB regulations and institutional guidelines. All procedures adhered to the ethical principles outlined in the Declaration of Helsinki.

### 2.2. The G-SCORE Strategy and the Bilateral Gum-Chewing (BGC) Protocol

**Figure 1:**
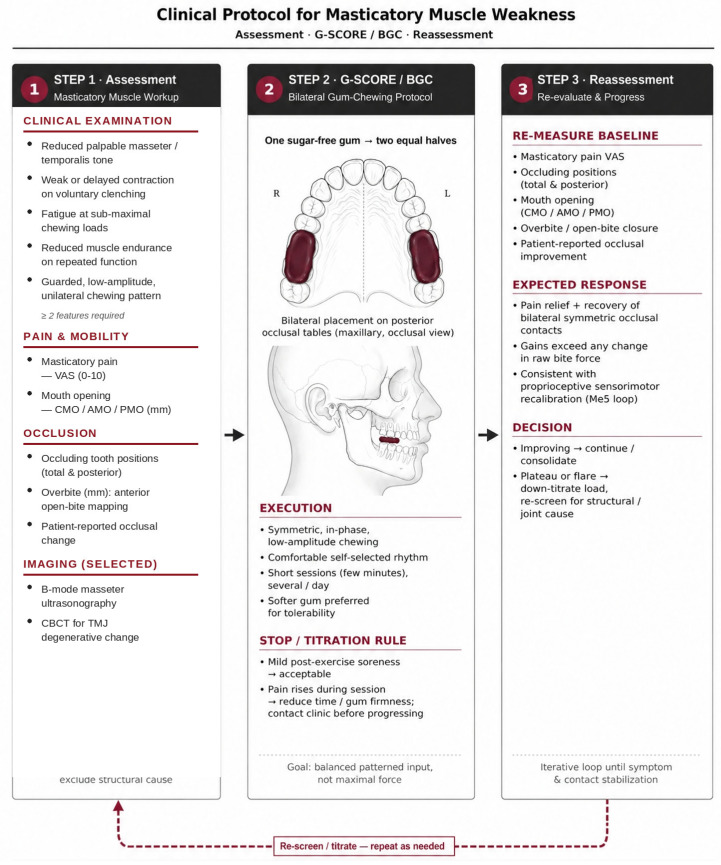
Operational workflow of the G-SCORE strategy delivered as the bilateral gum-chewing (BGC) protocol. Step 1—assessment of the masticatory muscle-weakness phenotype (clinical examination, pain and mobility, occlusal mapping, and selected imaging) and exclusion of a structural cause. Step 2—the prescribed BGC protocol: one sugar-free gum split into two equal halves placed simultaneously on both posterior occlusal tables and chewed symmetrically in short, self-titrated sessions, with a defined stop/titration rule. Step 3—reassessment of the same outcome items, with the expected response dominated by pain relief and recovery of bilateral symmetric occlusal contact rather than raw bite force; the process iterates with re-screening and load titration as needed. *Red-shaded objects in Step 2 depict the chewing gum in its bilateral placement; solid arrows indicate the sequential progression across Steps 1–3, and the dashed arrow denotes the iterative re-screening/titration loop*.

G-SCORE is a conceptual strategy: it reframes chewing-based exercise in this phenotype as proprioceptive sensorimotor calibration of occlusion driven by rhythmic, balanced functional loading, rather than as muscle strengthening. In clinical practice the strategy is operationalized through the BGC protocol, summarized in Figure 1 and detailed here.

A single piece of commercially available sugar-free chewing gum is divided into two equal halves. The patient places one half over the posterior occlusal table of each side simultaneously, so that the right and left masseter and temporalis are loaded symmetrically and in phase. This bilateral, balanced placement is the defining feature of the BGC protocol: it deliberately counteracts the unilateral, pain-avoidant chewing pattern that these patients commonly adopt, and it delivers equal proprioceptive loading to both dental arches, which a single unilateral gum bolus cannot—an argument also advanced for bilateral, rather than unilateral, chewing exercise [1]. A softer gum is preferred, to keep the task tolerable and to avoid the transient pain and fatigue associated with very hard gum [15].

Chewing is performed at a comfortable, self-selected rhythm in short, time-limited sessions (on the order of a few minutes), repeated a small number of times per day, with load and duration titrated by the patient against symptoms. The governing principle is symmetric, low-amplitude, time-limited functional chewing rather than maximal clenching against a bite block: the goal is patterned, balanced sensorimotor input and tolerance, not maximal force production. A simple stop rule is applied—if pain increases during a session (as opposed to mild, transient post-exercise soreness that resolves), the patient reduces the duration or the gum firmness and resumes at a lower load, contacting the clinic before progressing further. When myalgic guarding is prominent at the outset, a short period of pain control and gentle range-of-motion and rest-position training precedes loaded chewing [4,12], after which bilateral chewing is introduced and progressed.

### 2.3. Neurophysiological Rationale: Trigeminal Proprioceptive Re-Education

The G-SCORE strategy is grounded in the proprioceptive organization of the trigeminal sensorimotor system rather than in muscle hypertrophy. Two classes of receptor dominate the relevant afferent supply. Periodontal mechanoreceptors, embedded among the collagen fibers of the periodontal ligament, are slowly adapting and encode the temporal, spatial, and intensive features of forces applied to the dentition; the central nervous system uses these signals for the moment-to-moment sensorimotor regulation of oral behavior, and subjects deprived of periodontal input lose the fine control of inter-dental forces [20,21]. Muscle spindles within the jaw-closing muscles signal muscle length, velocity, and acceleration, while joint receptors of the temporomandibular joint contribute position and movement information [22,23]. Together, these afferents furnish the brainstem with a continuous, bilaterally balanced picture of where the teeth meet and how the elevator muscles are loaded.

The primary afferents from the jaw-closing muscle spindles and from many periodontal receptors are unusual in that their cell bodies lie within the central nervous system, in the trigeminal mesencephalic nucleus (Me5) [24]. Me5 neurons project both to the trigeminal motor nucleus—forming the afferent limb of the jaw-jerk and load-compensation reflexes—and to trigeminal sensory territories including the principal (Pr5) and spinal (Sp5) nuclei, and they send ascending projections toward the thalamus and hypothalamus [24]. This architecture places Me5 at the center of the integration of jaw movement with proprioception during mastication, and its extra-trigeminal connections to the hypothalamus provide a route by which the rhythm of chewing can modulate higher-order state, including dopaminergic and satiety-related circuitry [24]. The same circuitry is sensitive to load and to pain: chronic masticatory pain and the protective behavior it provokes reduce and imbalance the afferent traffic through this loop.

On this basis we propose a self-reinforcing cycle in the patients described here: masticatory pain promotes chewing avoidance and a guarded, unilateral pattern, which reduces and de-synchronizes periodontal and spindle afferent input; degraded and asymmetric proprioceptive drive through Me5 in turn destabilizes the central representation of occlusion and the reflex regulation of elevator-muscle balance, which the patient experiences as the bite “not fitting,” reinforcing further avoidance. The BGC protocol is designed to interrupt this cycle. Bilateral, rhythmic, low-load gum chewing restores a balanced, patterned afferent barrage from periodontal receptors and muscle spindles on both sides, re-engaging the trigeminal loop and driving recalibration of the central representation of occlusion and of the symmetric force vectors that stabilize it—the calibration that gives G-SCORE its name; the rhythmic ascending drive may additionally favor pain modulation and affective stabilization, and repetitive, predictable, non-noxious loading is a plausible counter-stimulus to maladaptive central sensitization, consistent with the analgesic effect attributed to load-tolerance gains in masticatory resistance training [6].

The strategy is therefore best understood as proprioceptive sensorimotor re-education. On this account the expected and clinically most valuable outcomes are relief of myalgic pain and recovery of symmetric occlusal contact, which need not be accompanied by a proportional rise in maximal bite force or by masseter hypertrophy. This prediction is directly testable against the outcome data reported below, and it aligns with the observation that gum-chewing training can improve occlusal function without changing masseter thickness [16]. It also delimits the indication: where acute joint derangement or a fixed structural lesion is the operative problem, loaded chewing may overload rather than recalibrate, so the technique is best reserved for the sub-acute to chronic phase once pain is at least partly controlled.

### 2.4. Outcome Assessment

Outcomes were abstracted from the clinical record at baseline (pre-treatment) and at follow-up (post-treatment), in line with the DC/TMD physical examination domains [3]. Masticatory pain was recorded on a visual analog scale (VAS, 0–10). Mandibular mobility comprises comfortable mouth opening (CMO), active (maximum unassisted) mouth opening (AMO), and passive (assisted) mouth opening (PMO), in millimeters (interincisal). Occlusal status was captured from the clinical occlusal map as the number of occluding tooth positions, both total and posterior, and as overbite (mm; negative values denote anterior open bite). Patient-reported occlusal change at baseline and patient-reported occlusal improvement at follow-up were each recorded as present or absent. Temporomandibular joint osteoarthritis was assessed on cone-beam computed tomography (CBCT) when available, and masticatory muscle status on B-mode masseter ultrasonography in selected patients, a method previously used to quantify masseter thickness in chewing-exercise studies [1,2].

### 2.5. Statistical Analysis

Statistical analyses were performed using IBM SPSS Statistics for Windows, version 30.0 (IBM Corp., Armonk, NY, USA). Continuous outcomes are summarized as mean (standard deviation). Given the small, variable paired samples and the ordinal nature of the VAS, within-subject pre–post changes were characterized using the Wilcoxon signed-rank test, with the effect size reported as r = Z/√*n* (interpreted as small ≈ 0.1, medium ≈ 0.3, large ≈ 0.5). Categorical outcomes are reported as counts and proportions. No imputation was performed; each comparison used all patients with both values available. The analyses are descriptive and within-subject; no between-group inference is made, and the reported *p*-values quantify the consistency of the observed within-subject change rather than test a confirmatory hypothesis. To address multiplicity transparently, Bonferroni-adjusted *p*-values were computed for the six pre-defined primary pre–post comparisons (pain, total and posterior occluding positions, comfortable and active mouth opening, and overbite in the anterior open-bite subgroup), using an adjusted threshold of α = 0.05/6 ≈ 0.0083, and are reported alongside the raw *p*-values and effect sizes in Table 1. Interpretation prioritizes the magnitude and consistency of the observed effects across related outcomes; the corrected *p*-values are provided as a conservative safeguard against overinterpretation.

## 3. Results

### 3.1. Patients

Group-level within-subject changes are summarized in Table 1, and response rates in Table 2.

### 3.2. Pain and Mandibular Mobility

Masticatory pain fell markedly, from 5.04 ± 2.90 to 0.67 ± 0.92 on the 0–10 scale (*n* = 27; *p* < 0.001; r = 0.87). Twenty-three of 27 paired patients (85%) achieved a reduction of at least two points, and 15 (56%) became pain-free. Comfortable and active mouth opening showed substantial within-subject gains (CMO +7.9 mm, *p* = 0.005; AMO +3.8 mm, *p* < 0.001), whereas passive opening, already near-normal at baseline, did not change materially (PMO +1.1 mm, *p* = 0.157)—a pattern indicating that the gains were in pain-limited functional opening rather than in the structural range of the joint. Pain reduction and CMO/AMO gains all remained robust under the Bonferroni-adjusted threshold (α = 0.0083; Table 1), whereas PMO did not, consistent with the interpretation above.

### 3.3. Occlusal-Contact Recovery and Open Bite

The number of occluding tooth positions increased from 4.00 ± 2.19 to 6.43 ± 2.08 (*n* = 28; *p* < 0.001; r = 0.82), and posterior occluding positions from 3.36 ± 1.79 to 5.82 ± 1.56 (*n* = 28; *p* < 0.001; r = 0.87); occlusal contacts increased in 25 of 28 paired patients (89%). In the six patients with a documented anterior open bite and paired data, overbite improved from −3.08 ± 1.74 to −1.17 ± 1.47 mm (*p* = 0.031; r = 0.88), with improvement in all six. These changes occurred without any occlusal adjustment, prosthetic alteration, or orthodontic intervention. Occluding-position recovery (total and posterior) remained robust under the Bonferroni-adjusted threshold; the anterior open-bite finding did not (adjusted *p* = 0.186) and is presented as a descriptive observation in a subgroup too small (*n* = 6) for confirmatory inference, interpreted accordingly in the Section 4.

### 3.4. Patient-Reported Occlusal Improvement

Of the 26 patients reporting an occlusal-change complaint at baseline, 13 explicitly reported occlusal improvement at follow-up; the remainder were not consistently re-questioned in the record. The recovery of bilateral posterior occlusal contact documented on the clinical occlusal maps is the plausible objective correlate of this perceived improvement, mirroring the group-level increase in occluding positions.

### 3.5. Previously Reported Illustrative Cases

Two of these patients have been described in detail in our earlier case reports [25,26] and are summarized only briefly here. In the first, a young woman developed masticatory pain and an anterior open bite after repeated masseter botulinum-toxin injection for aesthetic masseter reduction; the bilateral gum-chewing protocol resolved her pain and progressively closed the anterior open bite to edge-to-edge contact, with no occlusal equilibration, prosthetic alteration, or orthodontics [25]. In the second, a 68-year-old woman developed a posterior open bite and occlusal change after long-term wear of a partial-coverage oral appliance for a temporomandibular disorder; bilateral gum-chewing exercise restored a stable occlusal relationship without any irreversible occlusal treatment [26]. Both cases reproduce the pattern emphasized here—pain relief and recovery of symmetric occlusal contact achieved through restored balanced functional loading rather than any change to the dentition.

## 4. Discussion

We introduce G-SCORE—a proprioceptive sensorimotor recalibration strategy delivered as the BGC protocol—for orofacial-pain patients in whom masticatory muscle weakness, myalgic pain, and perceived occlusal change coexist, and characterize within-subject changes in a retrospective series of 41 patients managed under this strategy. The findings are interpreted below in five steps: the pattern of response and its mechanistic reading, the distinct phenotype the technique addresses, the clinical implication for conservative management, alternative explanations that the design cannot exclude, and the limitations that frame these observations.

### 4.1. Pattern of Response Favors Sensorimotor Recalibration over Strengthening

The hierarchy of effects is the central observation. The largest changes were the fall in masticatory pain (r = 0.87) and the recovery of total and posterior occluding positions (r = 0.82 and 0.87), accompanied by concordant gains in pain-limited mouth opening but no change in passive opening. These are outcomes of coordination and balanced occlusal contact rather than of raw force, and in a relatively young cohort (mean age 39.5 years) age-related sarcopenia is unlikely to be the operative mechanism [8,9]. The dissociation—functional and occlusal recovery without a commensurate change in force—is precisely what a trigeminal sensorimotor model predicts, and what the gum-chewing literature has independently reported: improved occlusal function without a measurable change in masseter thickness [16] and increases in voluntary bite force that may reflect motor learning rather than hypertrophy [19].

A recalibration reading is further supported by the afferent dependence of masticatory motor output. Masticatory muscle activity is shaped continuously by sensory input from the dentition and the food bolus, such that altering textural and loading cues measurably changes muscle recruitment and chewing pattern [27]; periodontal mechanoreceptors and muscle spindles, relayed through the trigeminal mesencephalic nucleus (Me5), encode the magnitude, direction, and timing of occlusal load and tune the motor command accordingly [20,21,22,24]. On this account, the recovery of bilateral posterior contact is the plausible objective correlate of the patients’ perceived occlusal improvement, achieved by restoring symmetric, rhythmic afferent input and balanced force vectors rather than by any change to the dentition. This is the calibration that the G-SCORE concept names.

### 4.2. A Distinct Patient Phenotype

The population differs from that in most masticatory-exercise studies. The established chewing-exercise literature addresses either healthy adults or frail older adults with oral hypofunction and uses force gain as the primary endpoint [1,2,16,17,18]; here the patients are predominantly younger, present with pain and a perceived occlusal disturbance, respond poorly to conventional conservative care, and were managed through a deliberately bilateral, low-load, symptom-titrated protocol rather than a force-maximizing one. The technique therefore occupies a gap between geriatric oral-function programs and conventional temporomandibular-disorder physiotherapy directed at hyperactive muscles [6,7].

The referral cohort is deliberately heterogeneous in etiology—temporomandibular joint osteoarthritis, post-splint or post-appliance settings, post-masseter botulinum-toxin exposure, and single cases following facial trauma, arthroplasty, and masseter-reduction surgery—and this heterogeneity is a legitimate methodological concern. The entry criterion, however, was not the referral etiology but the shared operational category of reduced masticatory muscle activation with pain on function and a perceived occlusal disturbance in a structurally stable dentition, ascertained by uniform expert orofacial-pain clinical examination (Section 2.1.2). Reduced muscle activation is the common downstream state on which the disparate upstream aetiologies converge in this series. On the proposed model, these settings share reduced, asymmetric periodontal and spindle afferent drive through the trigeminal mesencephalic loop, so treating them as a single phenotype is a testable prediction of the framework rather than an analytic oversight; if the model is correct, a common intervention should produce a common pattern of response despite different upstream aetiologies, which is what the present data show. Formal etiology-stratified inference is nevertheless not attempted here because each subgroup is too small to sustain it, and subgroup-stratified prospective evaluation is a defined next step.

### 4.3. Clinical Implication: Avoiding Irreversible Occlusal Treatment

By restoring perceived and measured occlusal contact without altering the dentition, the technique may allow selected patients to avoid irreversible occlusal treatment—equilibration, extensive restoration, or orthodontics—undertaken to chase a perceived bite change that is, on this account, of neuromuscular rather than structural origin. This is consistent with the principle that reversible, conservative strategies are the appropriate first line for temporomandibular disorders [4,5], and with the broader reappraisal that finds little ground for a causal role of dental occlusion in their pathophysiology [28]. The implication is particularly relevant after botulinum-toxin or masseter-reduction procedures, which themselves reduce bite force and masseter thickness [14], and after prolonged splint or oral-appliance wear—settings represented in the present series and in our earlier case reports (Section 3.5) [25,26]—where the perceived occlusal change is plausibly a downstream effect of altered muscle loading and afferent input. From a practical standpoint, the BGC protocol requires no equipment beyond commercially available sugar-free gum, can be prescribed by a general dental practitioner as well as by an orofacial-pain specialist, is reversible, and carries no known risk of iatrogenic occlusal change; where it fails to produce the expected pattern of response after an adequate trial, the patient can be re-triaged to standard care without having been committed to any irreversible procedure. On this basis, a trial of the BGC protocol may be reasonable to consider before any irreversible occlusal step is planned in this specific phenotype, subject to the mechanistic caveats set out below.

### 4.4. Alternative Explanations

The observed changes are open to several non-mechanistic readings that an uncontrolled retrospective design cannot exclude, and these merit explicit consideration. First, natural history and regression to the mean can produce a fall in pain scores in patients who present at a peak of severity, and the retrospective design cannot separate this from a true treatment effect. Second, concurrent conservative care—rest-position training, patient education, home care, and pharmacological analgesia in a subset of cases—may have contributed independently to the improvement, particularly in the pain domain. Third, a non-specific chewing-exercise effect—simply reloading the elevator muscles, whether or not the loading is bilaterally symmetric—could account for part of the recovery in occlusal contact and mouth opening without invoking proprioceptive recalibration specifically. Fourth, a reduction in pain-avoidance behavior, once the patient is explicitly instructed to reload the dentition in a structured way, may produce measurable functional gain by removing a behavioral suppressor of chewing rather than by altering the underlying sensorimotor circuitry. Fifth, voluntary, effort-dependent measures are subject to learning effects on repeated testing [19]; part of any change in mouth opening—and, had it been systematically assessed, in bite force—could reflect this rather than a mechanistic gain.

The pattern of response argues against any one of these accounts being sufficient. The largest gains were in pain and in objectively documented bilateral occlusal-contact recovery, with concordant improvement in pain-limited but not passive mouth opening; this hierarchy is more compatible with a sensorimotor-recalibration mechanism than with pure natural history, non-specific loading, or a learning effect on effort-dependent measures. Nevertheless, none of these alternatives can be excluded on the present data, and a definitive mechanistic attribution requires the prospective controlled design outlined in Section 4.5.

### 4.5. Limitations and Future Directions

The design is retrospective, single-center, and uncontrolled, so within-subject improvement cannot be separated from natural history, concurrent conservative care, or learning effects on effort-dependent measures, since voluntary bite force and mouth opening can rise with training in the absence of true strengthening [19]. The proposed proprioceptive-recalibration mechanism is grounded in the neuroanatomy of the trigeminal mesencephalic loop and consistent with the observed hierarchy of response [16,19,20,21,22,23,24], but no direct neurophysiological measure was obtained; it is therefore compatible with, but not proven by, the present data. Documentation was incomplete and the paired sample differs by outcome; masseter ultrasonography and bite-force measurement were performed in a subset only (12 and a smaller subset, respectively) and served to corroborate the clinical phenotype rather than establish it (Section 2.1.2). The cohort is aetiologically heterogeneous (Section 4.2), and individual subgroup sizes preclude formal stratified inference; the anterior open-bite finding (*n* = 6) and passive mouth opening did not remain significant after Bonferroni correction (Table 1) and should be interpreted with corresponding caution.

Future work should build on this technique through a prospective controlled trial with standardized dosing, a defined comparator (e.g., unilateral chewing or usual conservative care), etiology-stratified enrolment, and blinded objective assessment of occlusal contact and force, together with neurophysiological read-outs—such as surface EMG and tactile-detection thresholds at the dentition—capable of testing the proprioceptive-recalibration hypothesis and the G-SCORE concept directly.

## 5. Conclusions

We introduce G-SCORE (Gum-chewing-mediated Sensorimotor Calibration of Occlusion through Rhythmic Exercise), delivered as a simple, low-cost, non-invasive bilateral gum-chewing (BGC) protocol, for patients with masticatory muscle weakness who present with masticatory pain and perceived occlusal change and who respond poorly to conventional conservative care. In a retrospective series of 41 patients, the protocol was accompanied by marked pain relief and recovery of bilateral occlusal contact—a pattern more compatible with proprioceptive sensorimotor re-education through the trigeminal mesencephalic loop [24] than with muscle strengthening. If confirmed on prospective controlled testing, this pattern would imply that a perceived occlusal disturbance in this phenotype can be addressed without altering the dentition, offering selected patients a reversible alternative to irreversible occlusal treatment. Because the design is retrospective and uncontrolled, the present observations are descriptive and require confirmation through prospective controlled trials with standardized dosing, defined comparators, and objective neuromuscular and mechanistic read-outs.

## Figures and Tables

**Table 1:** Paired pre- versus post-treatment outcomes (Wilcoxon signed-rank test). Negative overbite denotes anterior open bite. Bonferroni-adjusted *p*-values are computed against a threshold of α = 0.05/6 ≈ 0.0083 for the six pre-defined primary comparisons (see Section 2.5).

Outcome (Unit)	*n*	Pre, Mean ± SD	Post, Mean ± SD	Raw *p*	Bonferroni-Adjusted *p*	r
Masticatory pain, VAS (0–10)	27	5.04 ± 2.90	0.67 ± 0.92	<0.001	<0.006 *	0.87
Occluding positions, total (*n*)	28	4.00 ± 2.19	6.43 ± 2.08	<0.001	<0.006 *	0.82
Posterior occluding positions (*n*)	28	3.36 ± 1.79	5.82 ± 1.56	<0.001	<0.006 *	0.87
Comfortable opening, CMO (mm)	14	36.7 ± 8.7	44.6 ± 5.5	0.005	0.030 *	0.82
Active opening, AMO (mm)	34	43.0 ± 7.0	46.8 ± 4.6	<0.001	<0.006 *	0.65
Passive opening, PMO (mm)	18	47.2 ± 5.5	48.3 ± 3.8	0.157	0.942	0.39
Overbite, open-bite subgroup (mm)	6	−3.08 ± 1.74	−1.17 ± 1.47	0.031	0.186	0.88

VAS, visual analog scale; CMO/AMO/PMO, comfortable/active/passive maximum mouth opening; SD, standard deviation. Wilcoxon signed-rank test; effect size r = Z/√*n*, interpreted as small (≈0.1), medium (≈0.3), or large (≈0.5) per Cohen’s convention. Bonferroni-adjusted *p*-values are calculated as raw *p* × 6 (the number of pre-defined primary comparisons), capped at 1.0. * remains robust under the Bonferroni-adjusted threshold α = 0.0083. The anterior open-bite finding (*n* = 6) is a subgroup analysis presented for descriptive purposes and should not be interpreted as confirmatory.

**Table 2:** Responder rates for the principal outcomes.

Response Criterion	Responders/*n*	%
Masticatory pain reduced by ≥2 VAS points	23/27	85
Became pain-free (post VAS = 0)	15/27	56
Increase in number of occluding positions	25/28	89
Anterior open bite reduced (subgroup)	6/6	100
Patient-reported occlusal improvement *	13/26	50

* Denominator is the 26 patients with a baseline occlusal-change complaint; the remainder were not consistently re-questioned in the record.

## Data Availability

The data supporting the findings of this study are available from the corresponding author upon reasonable request.

## Abbreviations

The following abbreviations are used in this manuscript:

AMO	Active (maximum unassisted) mouth opening
BGC	Bilateral gum-chewing
CBCT	Cone-beam computed tomography
CMO	Comfortable mouth opening
DC/TMD	Diagnostic Criteria for Temporomandibular Disorders
G-SCORE	Gum-chewing-mediated Sensorimotor Calibration of Occlusion through Rhythmic Exercise
Me5	Trigeminal mesencephalic nucleus
PMO	Passive (assisted) mouth opening
Pr5	Trigeminal principal sensory nucleus
RDC/TMD	Research Diagnostic Criteria for Temporomandibular Disorders
SD	Standard deviation
Sp5	Trigeminal spinal (sensory) nucleus
VAS	Visual analog scale
